# Kudzu Resistant Starch: An Effective Regulator of Type 2 Diabetes Mellitus

**DOI:** 10.1155/2021/4448048

**Published:** 2021-10-13

**Authors:** Xinqi Song, Huanhuan Dong, Zhenzhong Zang, Wenting Wu, Weifeng Zhu, Hua Zhang, Yongmei Guan

**Affiliations:** ^1^Key Laboratory of Modern Preparation of Traditional Chinese Medicines, Ministry of Education, Jiangxi University of Chinese Medicine, 330004 Nanchang, China; ^2^School of Pharmacy, Jiangxi University of Chinese Medicine, 330004 Nanchang, China

## Abstract

Kudzu is a traditional medicinal dietary supplement, and recent research has shown its significant benefits in the prevention/treatment of type 2 diabetes mellitus (T2DM). Starch is one of the main substances in Kudzu that contribute decisively to the treatment of T2DM. However, the underlying mechanism of the hypoglycemic activity is not clear. In this study, the effect of Kudzu resistant starch supplementation on the insulin resistance, gut physical barrier, and gut microbiota was investigated in T2DM mice. The result showed that Kudzu resistant starch could significantly decrease the value of fasting blood glucose and the levels of total cholesterol, total triglyceride, and high-density lipoprotein, as well as low-density lipoprotein, in the blood of T2DM mice. The insulin signaling sensitivity in liver tissue was analyzed; the result indicated that intake of different doses of Kudzu resistant starch can help restore the expression of IRS-1, p-PI3K, p-Akt, and Glut4 and thus enhance the efficiency of insulin synthesis. Furthermore, the intestinal microorganism changes before and after ingestion of Kudzu resistant starch were also analyzed; the result revealed that supplementation of KRS helps to alleviate and improve the dysbiosis of the gut microbiota caused by T2DM. These results validated that Kudzu resistant starch could improve the glucose sensitivity of T2DM mice by modulating IRS-1/PI3K/AKT/Glut4 signaling transduction. Kudzu resistant starch can be used as a promising prebiotic, and it also has beneficial effects on the gut microbiota structure of T2DM mice.

## 1. Introduction

Kudzu (Pueraria lobata) is a popular traditional Chinese medicine and a medicinal vegetable widely distributed in subtropical and temperate regions such as Southern China, Japan, and Vietnam [1, 2]. The active components and pharmacological effects of Kudzu have received widespread attention [3, 4]. It is commonly used for the treatment of hyperglycemia, hyperlipidemia, cancer, and cardiovascular diseases due to the presence of therapeutically active components such as isoflavones, saponins, and starch [5]. Among the functional components, Kudzu starch is commonly used as a functional food to manage type 2 diabetes mellitus (T2DM). Kudzu starch contains a certain amount of resistant starch, which is a new type of dietary fiber frequently reported in recent studies, and a large number of studies have shown that resistant starch is an important part of daily food with significant hypoglycemic properties [6].

There is no doubt that the development of T2DM is a direct threat to human health [7, 8]. Increasing evidence has shown that changes in dietary structure can significantly affect or hinder the process of T2DM [9]. Thus, healthier dietary habits, especially a greater intake of healthy natural products with low calories and low glycemic index without adverse effects, have become a major trend for controlling T2DM [10]. Numerous studies have demonstrated the specific role of resistant starch in the maintenance of normal physiological function with a focus on the prevention/treatment of T2DM [11]. Resistant starch cannot be digested in the small intestine but can be fermented in the colon [12]. And furthermore, it is used as a functional product to lower glucose, gradually replacing traditional starch products.

Although the precise mechanisms of the hypoglycemic activity of resistant starch have not been fully elucidated, there is a significant positive correlation between the consumption of foods rich in resistant starch and improvement in hyperglycemic status [13]. One hypothesis suggests that the mechanism may involve modulating the associated gut microbial structure and improving intestinal inflammation thereby inhibiting the development of T2DM. For instance, rice flour enriched with resistant starch has been shown to significantly suppress chronic inflammation associated with diabetes and obesity-induced intestinal disorders in mice by increasing the expression levels of GPR41 and GPR43 proteins, reducing fecal pH values and inflammatory factor levels in diabetic mice, while increasing the content of probiotics such as *Bifidobacterium* and *Lactobacillus* [14]. In addition, high-amylose resistant starch might exert beneficial effects on intestinal and systemic parameters by increasing cecum weight as well as the amount of intestinal short-chain fatty acids and decreasing pH reduction in intestinal content, while improving the gene expression for gluconeogenesis and barrier function [15].

Furthermore, T2DM patients often develop insulin resistance, which is mainly manifested by abnormal insulin signals in the liver and muscles [16]. Therefore, insulin signaling pathways play a critical role in the prevention and treatment of T2DM. For instance, a resistant starch-rich brown rice had been shown to prevent T2DM and hypertriglyceridemia by regulating the expression of glucose metabolism gene expression in the liver [17]. Lotus seed resistant starch had also been reported to exhibit significant hypoglycemic effects by regulating the expression levels of genes including insulin secretion, insulin signaling, and metabolism-related signaling pathways [18]. The hypoglycemic process of resistant starch may occur through multiple signaling pathways. The IRS-1/PI3K/Akt/Glut4 signaling pathway is a classical insulin signaling pathway that plays an influential role in alleviating diabetes-induced insulin resistance [19]. Therefore, studying the expression of protein levels related to the IRS-1/PI3K/Akt/Glut4 pathway may help to elucidate the hypoglycemic mechanism of resistant starch.

Kudzu resistant starch (KRS) can be produced by extracting and purifying or modifying and processing from Kudzu starch, which has the properties of resistant starch, including good hypoglycemic and hypolipidemic functions. However, the exact mechanisms related to hypoglycemic activity, anti-inflammation, and gut microbiota improvement have not been fully elucidated. In this study, we explored the hypoglycemic activity of KRS in T2DM mice by monitoring diabetic indicators and changes in the intestinal microecological environment. Meanwhile, the molecular mechanism of hypoglycemia was also determined by measuring the expression of the IRS-1/PI3K/AKT/Glut4 pathway.

## 2. Materials and Methods

### 2.1. Preparation of Kudzu Resistant Starch (KRS)

Kudzu starch was purchased from Xinjian, Jiangxi Province, China. The kudzu starch suspension (15%, based on dry starch) was heated at 90°C in a water bath for 30 min while stirring. The starch was heated into a paste, treated at 121°C for 30 min, and then cooled to room temperature. The citrate buffer (0.135 mol/L) was used to adjust the pH to 4.5, followed by the reaction between pullulanase (60 U/g) and the suspension for 4 h. After that, the starch suspension was inactivated at high temperature and cooled to room temperature, then aged 24 h under refrigeration, dried at 60°C for 12 h, and then crushed and sieved. The treated starch was prepared with citric acid buffer (0.135 M, pH =2). Then, 10 mL of pepsin (400 U/mL) was added to 15% starch milk, stirred in a water bath at 40°C, and adjusted the pH to 7; then, 10 mL of high-temperature-resistant *α*-amylase (400 U/mL) was added, stirred in a water bath at 95°C for 30 min, adjusted the pH to 4.5, added 0.6 g of glycosylase (100000 U/g), and stirred in a water bath at 60°C for 1 h. The enzyme was inactivated, and the supernatant was removed after repeated washing with 95% ethanol and stirred in the water bath at 60°C for 1 h. After repeated washing, the supernatant was removed, dried at 60°C for 12 h, crushed, and sieved, and the KRS was obtained.

### 2.2. Experimental Animals

A total of 60 healthy C57BL/6J male mice (18~22 g) were provided by Hunan SJA Laboratory Animal Co. and housed in a controlled temperature (22 ± 2°C) and humidity (60 ± 10%) under a 12/12 h light/dark cycle with ad libitum access to food and water in the Animal Center of Jiangxi University of Chinese Medicine (Nanchang, Jiangxi, China). This animal experiment was reviewed and approved by the Ethics Committee of Jiangxi University of Chinese Medicine for experimental animals.

### 2.3. Experimental Design

**Figure 1:**
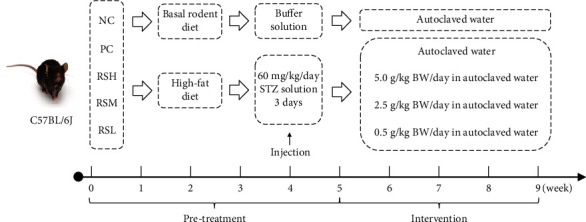
Experimental groups and respective treatments.

Experimental groups and respective treatments are described in Figure 1. After 1 week of acclimatization, all mice were randomly divided into a normal control (NC) group (10 mice, fed with basal rodent diet, energy of 3450 kcal/kg) and high-fat feeding group (50 mice, fed with high-fat diet; the high-fat diet: basal diet 63.6%, cholesterol 1.2%, sodium salt 0.2%, egg yolk powder 10%, sucrose 15%, and lard 10%; energy of 4343.2 kcal/kg). After 4 weeks of rearing, mice from the high-fat diet feeding group were repeatedly injected with fresh STZ solution (60 mg/kg BW, on 3 consecutive days) (Sigma-Aldrich, St Louis, MO, USA), and mice from the NC group were injected with saline in equal volume [20]. After 4 days, 50 diabetic mice were monitored for fasting blood glucose, and mice with fasting blood glucose greater than 11.1 mmol/L were identified as T2DM mice for this research. T2DM mice were randomly divided into 5 groups (*n* = 10) as follows: positive control (PC) group: treated with autoclaved water; high-dose resistant starch (RSH) group: treated with 5.0 g/kg BW/day in autoclaved water; medium-dose resistant starch (RSM) group: treated with 2.5 g/kg BW/day in autoclaved water; and low-dose resistant starch (RSL) group: treated with 0.5 g/kg BW/day in autoclaved water [21]. The mice in the NC group were supplemented with the same volume of autoclaved water.

During the experiment, the body weight and fasting blood glucose were recorded weekly. After 4 weeks of intragastric administration, mice were fasted for 12 h before being sacrificed, and the blood and fecal samples and liver and colon tissues were collected and stored at −80°C for further analysis.

### 2.4. Determination of Fasting Blood Glucose (FBG), Body Weight, and Oral Glucose Tolerance Test (OGTT)

During the experiment, the body weight and FBG were recorded weekly. FBG was measured by One-Touch Glucose Monitor (LifeScan, PA, USA) in each mouse after blood sample collection via tail scoring, and mice were fasted for 6 h before measurement. Six mice per group were randomized to an oral glucose tolerance test the day before the end of the administration. Mice were gavaged with glucose (2 g/kg BW) dissolved in autoclaved water after 12 h fasting, and blood glucose value was measured at 0, 30, 60, 90, and 120 min. Finally, AUC value was calculated.

### 2.5. Determination of Serum Lipids

According to the kit instructions (Nanjing Jiancheng Biochemistry Co., Beijing, China), the levels of triglycerides (TG), total cholesterol (TC), low-density lipoprotein cholesterol (LDL-C), and high-density lipoprotein cholesterol (HDL-C) were determined.

### 2.6. Determination of Serum Insulin Levels and Assessment of Insulin Resistance

In the serum, the blood insulin of all groups was determined by ELISA kits (Jiangsu Mmbio Co., Ltd.). Homeostasis model assessment-insulin resistance (HOMA-IR) was calculated by using the formula:

(1) $$\begin{array}{c} \text{HOMA}-\text{IR}=\frac{\left(\text{insulin}\times \text{FBG}\right)}{22.5}. \end{array}$$

### 2.7. Determination of Inflammatory Factor

The inflammatory factor levels of monocyte chemotactic protein-1 (MCP-1) and tumor necrosis factor-alpha (TNF-*α*) were determined in the serum according to the ELISA kits (Jiangsu Mmbio Co., Ltd.).

### 2.8. Intestinal Permeability Assessment

Before the end of the experiment, intestinal permeability assay was performed by administration of D-mannitol [22]. All mice were gavaged with 150 *μ*L D-mannitol solution (0.6 g/kg BW) precisely after fasting without water for 6 h before the experiment. The serum was collected for ELISA and recorded for intestinal permeability index. In addition, the cytokine levels of lipopolysaccharide (LPS) content were determined with blood samples collected before the end of the experiment following the instructions of the ELISA kit (Jiangsu Mmbio Co., Ltd.).

### 2.9. Western Blot Analysis

The gene expression of insulin receptor substrate 1 (IRS-1), protein kinase B (Akt), phosphorylated protein kinase B (p-Akt), phosphatidylinositol-3-kinase (PI3K), phosphorylated phosphatidylinositol-3-kinase (p-PI3K), glucose transporter 4 (Glut4), zonula occludens-1 (ZO-1), and occludens at the protein level was assayed by western blotting. Liver and colon tissue lysates were centrifuged at 4°C and 12,000 × g for 15 min. The supernatant was collected, and the protein concentration was determined using the BCA protein quantification kit and then stored at -80°C for further analysis. According to the protein quantification results, the required protein is added to the appropriate amount of loading buffer, and the prepared PAGE gel is subjected to electrophoresis. Subsequently, proteins are transferred to PVDF membranes using a semidry transfer, which can be tested for success with Rizin Red staining. The membranes are closed with 5% skim milk for 1 h or overnight at 4°C and then incubated with diluted primary antibody for 1 h at room temperature or overnight at 4°C. After TBST washing, the HRP-labeled secondary antibody is diluted at 1 : 1000 and incubated with the membrane for 1 h at 37°C. The membrane is washed 3 times with TPST for 5 min each. Then, the final blot was displayed by ECL assay for the desired blot.

### 2.10. RNA Isolation and Real-Time RT-PCR

Total RNA was extracted from colon tissue using the TRIzol reagent and real-time quantitative PCR was carried out by the RT-PCR detection system (ABI, USA).  Table 1 lists the sequence of the primers used. Relative quantification was calculated using the comparative 2^−*ΔΔ*Ct^ method. GAPDH was used as an internal control in this study [23].

### 2.11. Determination of Fecal Short-Chain Fatty Acid Content

Fecal short-chain fatty acid contents were determined by Gas Chromatography-Mass spectrometry (GC-MS). Disperse 200 mg of feces in 1000 *μ*L of 0.005 M NaOH aqueous solution and add 10 *μ*L of 5 mg/mL of 2-ethylbutyric acid dilution (dissolved in 0.005 M NaOH) as internal standard. Mix and centrifuge, take 500 *μ*L of supernatant into a 10 mL centrifuge tube, and add 300 *μ*L of pure water, 300 *μ*L of isopropanol, 200 *μ*L of pyridine, and 100 *μ*L of PCF (propyl chloroformate) solution for derivatization. After extracting the derivatized material with hexane, add 300 *μ*L of hexane, mix, and centrifuge the supernatant into a new 2 mL centrifuge tube. Add 200 *μ*L and repeat the previous step, then combine the supernatants, mix, and centrifuge the supernatants for GC/MS. The injector, ion source, and detector temperatures were maintained at 280°C, 230°C, and 150°C, respectively. After an initial period of 4 min at 50°C, the temperature was increased to 70°C for 1 min at a rate of 10°C/min, then increased to 85°C for 1 min at a rate of 10°C/min, and followed by a 5°C/min increase to 110°C for 1 min. In the last step, the oven temperature was increased by 30°C/min and maintained at 260°C. Then, the retention times were compared with the previous standards to determine the peak values.

### 2.12. Gut Microbiota Analysis

OTU table was generated and filtered according to the data volume; USEARCH (version 10.0) was used to cluster sequences at the 97% similarity level and by default filter OTUs at a threshold of 0.005% of the number of all sequences sequenced. QIIME software was used for alpha diversity and microbial taxon distribution analysis. Metastats statistical algorithm 24 is used in Mothur software to compare and test the difference of sequence quantity (i.e., absolute abundance) between phylum and genus levels of various classification units in groups. R software was used to analyze the composition structure of samples at the genus level, cluster the top 20 genera in abundance, and draw the heat map. Beta diversity analysis was mainly based on PCoA and NMDS analysis.

### 2.13. Statistical Analysis

Statistical data were expressed as means ± standard errors (SEM) and analyzed using SPSS software (version 19.0 SPSS Inc., Chicago, IL, USA) by one-way ANOVA. All results were generated using GraphPad Prism 8.0 software (GraphPad Software, San Diego, CA, USA). A level of *P* < 0.05 was considered to be statistically significant.

## 3. Results

### 3.1. Effect of KRS on Body Weight in T2DM Mice

**Figure 2:**
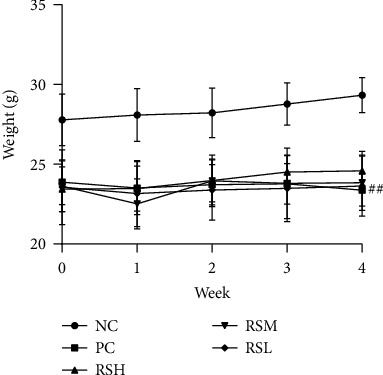
Effects of KRS on body weight in T2DM mice. Data are presented as mean ± SEM (*n* = 10). ^#^*P* < 0.05 and ^##^*P* < 0.01 as compared with the NC group. ^∗^*P* < 0.05 and ^∗∗^*P* < 0.01 as compared with the PC group.

The body weight changes of each group in 4 weeks are shown in Figure 2. The results showed that mice in the T2DM model group had significantly suppressed body weight gain (*P* < 0.05) and slow growth rate, which is typical of the T2DM syndrome. The KRS-treated group had a higher weight growth rate than the PC group, indicating a significant improvement in the typical T2DM syndrome.

### 3.2. Effect of KRS on Glucose Metabolism in T2DM Mice

**Figure 3:**
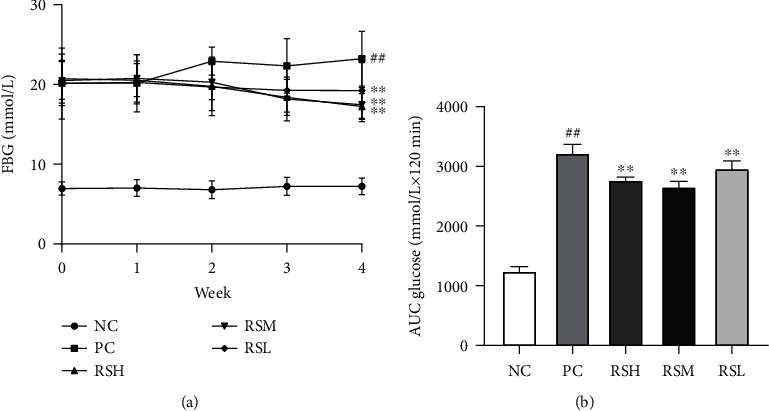
Effects of KRS on blood glucose profile, including FBG (a) and OGTT (b), in T2DM mice. Data of FBG (*n* = 10) and AUC glucose (*n* = 6) are presented as mean ± SEM. ^#^*P* < 0.05 and ^##^*P* < 0.01 as compared with the NC group. ^∗^*P* < 0.05 and ^∗∗^*P* < 0.01 as compared with the PC group.

As shown in Figure 3(a), the FBG levels of the mice in the NC group were maintained at 6.9-7.2 mmol/L, which were stable within the normal range, while the initial values of blood glucose in T2DM mice after STZ induction were all higher than 20 mmol/L, showing the symptoms of hyperglycemia in T2DM. The blood glucose values of the positive control mice were maintained at a high level and showed a slightly increasing trend. T2DM mice in the KRS-treated groups had significantly lower FBG levels after 2-week treatment than the PC group. At week 4, the KRS-treated groups showed highly significant differences from the PC group (*P* < 0.01). Different doses of KRS all showed good hypoglycemic efficacy in T2DM mice, and the hypoglycemic effect was better in the RSH and RSM groups. OGTT was used to determine the metabolic regulation of blood glucose and insulin synthesis function of the organism. As shown in the result in Figure 3(b), compared with the PC group, KRS treatment effectively inhibited OGTT. The area under the curve (AUC) could reflect the degree of glucose tolerance in each group of mice. Obviously, the high-dose KRS-treated and medium-dose-treated group had a lower AUC value than the positive control (*P* < 0.01). These results demonstrated the therapeutic potential of KRS in T2DM.

### 3.3. Effect of KRS on Blood Lipid Metabolism in T2DM Mice

**Figure 4:**
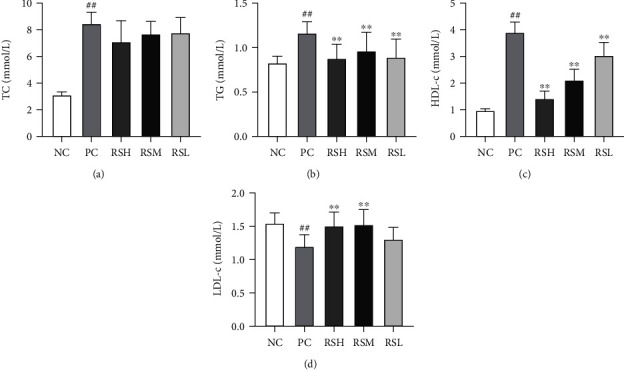
Effects of KRS on TC (a), TG (b), HDL-c (c), and LDL-C (d) in T2DM mice. Data are presented as mean ± SEM (*n* = 10). ^#^*P* < 0.05 and ^##^*P* < 0.01 as compared with the NC group. ^∗^*P* < 0.05 and ^∗∗^*P* < 0.01 as compared with the PC group.

The modulation of lipid levels in T2DM mice by KRS can be found in Figure 4. The abnormal lipid metabolism in T2DM is characterized by higher serum TC, TG, and LDL-C level along with lower serum HDL-C levels. Compared with the NC group, the levels of TG, TC, and LDL-C in the PC group were significantly increased (*P* < 0.01), while the HDL-C levels were significantly decreased (*P* < 0.01). Compared with the PC group, the KRS-treated mice in each group showed different degrees of improvement in lipid profile at the end of the experiment. TG and LDL-C levels were significantly reduced in all groups (*P* < 0.01), and TC levels were decreased in each group after treatment, but there was no significant difference (*P* > 0.05). In addition, the level of HDL-C in the RSH and RSM groups was significantly increased (*P* < 0.01) and recovered to the normal standards. The results indicated that KRS could improve the abnormal lipid metabolism in T2DM mice.

### 3.4. Effect of KRS on Serum Insulin Levels and Assessment of Insulin Resistance in T2DM Mice

**Figure 5:**
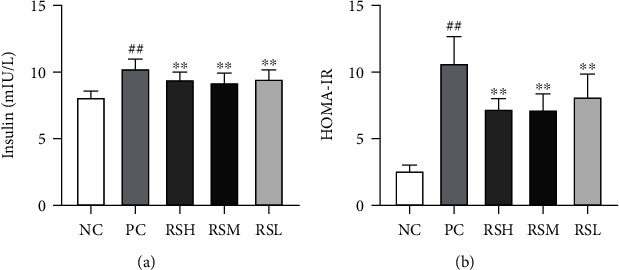
Effects of KRS on insulin (a) and HOMA-IR (b) in T2DM mice. Data are presented as mean ± SEM (*n* = 10). ^#^*P* < 0.05 and ^##^*P* < 0.01 as compared with the NC group. ^∗^*P* < 0.05 and ^∗∗^*P* < 0.01 as compared with the PC group.

As shown in Figure 5, the serum insulin levels of mice in the PC group were all higher than those in the NC group (*P* < 0.01), indicating the existence of significant abnormal insulin secretion in T2DM mice. The administration of different concentrations of KRS resulted in a significant decrease in serum insulin levels, which may account for the increased insulin sensitivity. The HOMA-IR results indicated that treatment with different doses of KRS significantly downregulated HOMA-IR compared with the PC group (*P* < 0.01). All the above results indicated that treatment of KRS for 4 weeks can effectively ameliorate insulin resistance in T2DM mice.

### 3.5. Effect of KRS on Inflammatory Factor in T2DM Mice

**Figure 6:**
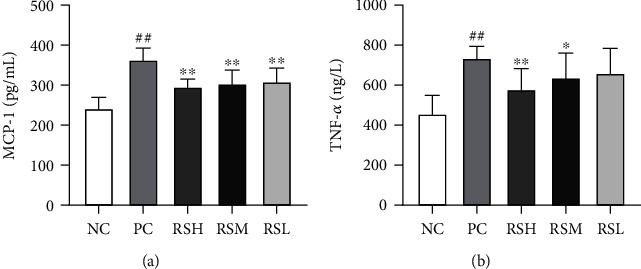
Effects of KRS on MCP-1 (a) and TNF-*α* (b) in T2DM mice. Data are presented as mean ± SEM (*n* = 10). ^#^*P* < 0.05 and ^##^*P* < 0.01 as compared with the NC group. ^∗^*P* < 0.05 and ^∗∗^*P* < 0.01 as compared with the PC group.

As shown in Figure 6, the levels of MCP-1 and TNF-*α* were significantly higher in the PC group compared with the NC group (*P* < 0.01), indicating a significantly higher level of inflammation in T2DM mice. After 4 weeks of treatment with different doses of KRS, the inflammation levels were all significantly reduced and showed a certain quantitative-effect relationship. Compared with the PC group, the MCP-1 level was significantly decreased in the KRS treatment groups (*P* < 0.01), and the TNF-*α* level was significantly decreased in the RSH group (*P* < 0.01). The results indicate that KRS treatment can effectively improve serum inflammatory factor levels in T2DM mice.

### 3.6. Effect of KRS on the IRS-1/PI3K/AKT/Glut4 Pathway in T2DM Mice

**Figure 7:**
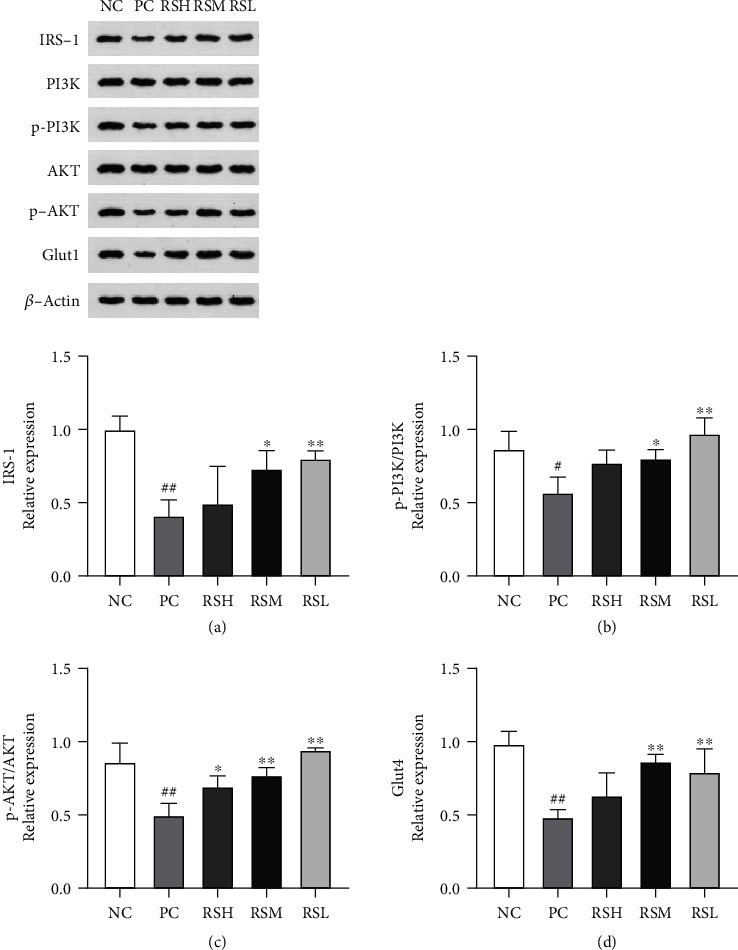
Effects of KRS on the gene expression at protein levels in T2DM mice: (a) IRS-1, (b) p-PI3K/PI3K, (c) p-Akt/Akt, and (d) Glut4. Data are presented as mean ± SEM (*n* = 10). ^#^*P* < 0.05 and ^##^*P* < 0.01 as compared with the NC group. ^∗^*P* < 0.05 and ^∗∗^*P* < 0.01 as compared with the PC group.

To evaluate the potential molecular mechanism of KRS to improve T2DM, key genes of the insulin signaling pathway including IRS-1, PI3K, p-PI3K, Akt, p-Akt, and Glut4 were investigated using western blotting. As shown in Figure 7, compared with the NC group, the relative expression levels of hepatic insulin signaling pathway proteins in the PC group mice were significantly changed, in which the relative expression of IRS-1, p-PI3K, p-Akt, and Glut4 proteins was significantly decreased (*P* < 0.01). The expression of related proteins increased after different doses of KRS treatment. In particular, in the RSM group, the relative expression of IRS-1 protein was increased (*P* < 0.05) and the relative expression of p-PI3K, p-Akt, and Glut4 protein was significantly increased (*P* < 0.01) compared with the PC group. The above results suggest that KRS can alleviate insulin resistance by regulating the expression of proteins related to the IRS-1/PI3K/AKT/Glut4 pathway.

### 3.7. Effect of KRS on Intestinal Permeability in T2DM Mice

**Figure 8:**
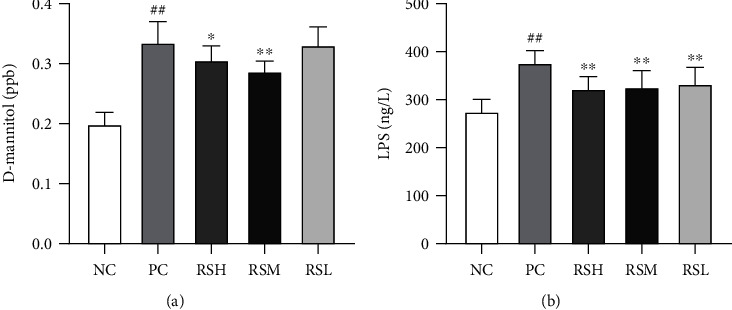
Effects of KRS on D-mannitol levels (a) and LPS (b) in T2DM mice. Data are presented as mean ± SEM (*n* = 10). ^#^*P* < 0.05 and ^##^*P* < 0.01 as compared with the NC group. ^∗^*P* < 0.05 and ^∗∗^*P* < 0.01 as compared with the PC group.

As shown in Figure 8, serum D-mannitol levels in mice in the PC group differed significantly (*P* < 0.01) compared to the NC group, and increased intestinal permeability led to increased serum D-mannitol levels. The serum D-mannitol levels were significantly lower in the RSM group compared to the PC group (*P* < 0.01). Compared with the NC group, the serum LPS content of mice in the PC group was significantly higher than that in the normal group (*P* < 0.01). Compared with the PC group, the serum LPS content of mice was reduced after KRS treatment (*P* < 0.01). The experimental results indicate that KRS can improve the repair of intestinal defense, and the medium dose of KRS can effectively alleviate the abnormal phenomenon of intestinal permeability.

### 3.8. Effect of KRS on Intestinal Tight Junction Protein and Mucin in T2DM Mice

**Figure 9:**
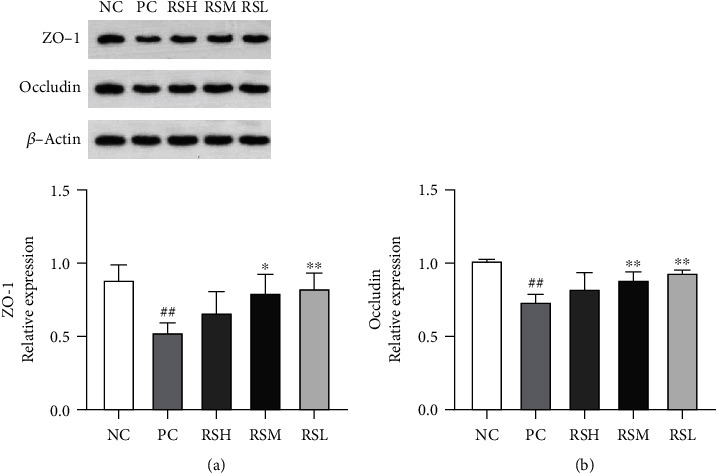
Effects of KRS on the gene expression at protein levels in T2DM mice: ZO-1 (a) and Occludin (b). Data are presented as mean ± SEM (*n* = 10). ^#^*P* < 0.05 and ^##^*P* < 0.01 as compared with the NC group; ^∗^*P* < 0.05 and ^∗∗^*P* < 0.01 as compared with the PC group.

**Figure 10:**
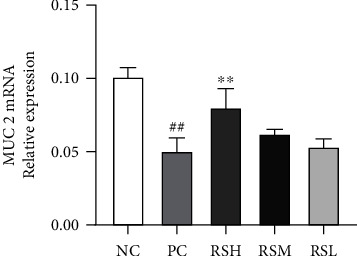
Effects of KRS on the MUC 2 mRNA expression in T2DM mice. Data are presented as mean ± SEM (*n* = 10). ^#^*P* < 0.05 and ^##^*P* < 0.01 as compared with the NC group. ^∗^*P* < 0.05 and ^∗∗^*P* < 0.01 as compared with the PC group.

As shown in Figure 9, the relative expression levels of ZO-1 and Occludin proteins in the PC group were significantly decreased compared with those in the NC group (*P* < 0.01), indicating that T2DM can directly affect the integrity of the intestinal mucosa. The relative expression of ZO-1 and Occludin protein in the colonic tissues of mice was increased after different doses of KRS treatment, with a highly significant increase in ZO-1 and Occludin protein expression in the RSL group (*P* < 0.01) and a significant increase in Occludin protein expression in the RSM group (*P* < 0.01). In contrast, there was no significant effect on the relative expression of ZO-1 and Occludin protein in the RSH group. As expected, as shown in Figure 10, the relative expression of MUC 2 mRNA in colonic tissues increased in all groups after KRS treatment compared with the PC group, with significant differences in the RSH group (*P* < 0.01). The results indicated that KRS could have a protective effect on the intestine.

### 3.9. Effect of KRS on Short-Chain Fatty Acids in T2DM Mice

**Figure 11:**
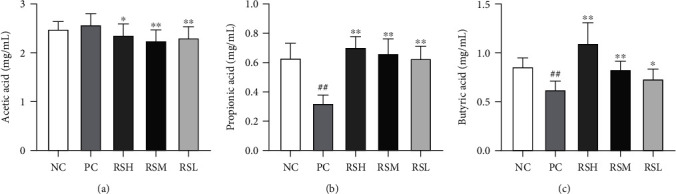
Effects of KRS on the concentration of SCFAs in T2DM mice: acetic acid (a), propionic acid (b), and n-butyric acid (c). Data are presented as mean ± SEM (*n* = 10). ^#^*P* < 0.05 and ^##^*P* < 0.01 as compared with the NC group; ^∗^*P* < 0.05 and ^∗∗^*P* < 0.01 as compared with the PC group.

The concentrations of acetic acid, propionic acid, and n-butyric acid are shown in Figure 11. Compared with the NC group, mice in the PC group showed a significant decrease (*P* < 0.01) in the concentrations of propionic acid and n-butyric acid and a slight increase in the concentration of acetic acid. The concentrations of acetic acid, propionic acid, and n-butyric acid in the feces of mice were varied after KRS treatment. In addition, mice in the RSH and RSM groups would produce more SCFAs, especially propionic acid and butyric acid, than mice in the RSL group. The results indicated that KRS can effectively regulate the concentration of fecal SCFAs in T2DM mice with the intake of KRS within the specified level.

### 3.10. Effect of KRS on Composition Analysis of Gut Microbiota in T2DM Mice

**Figure 12:**
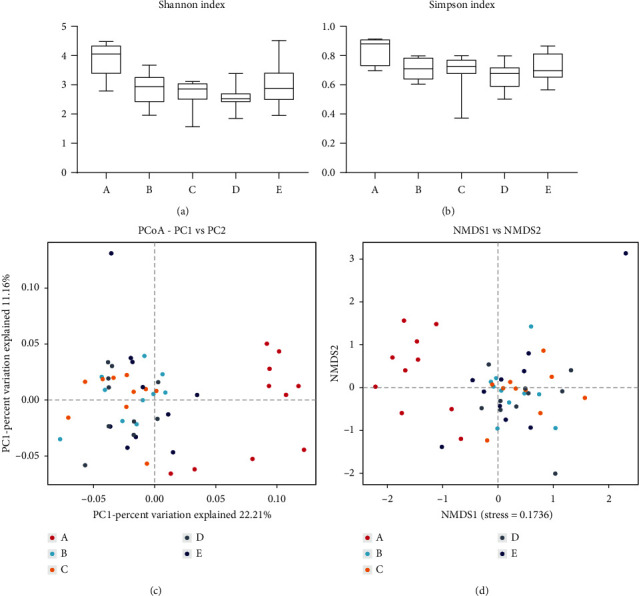
Comparison of richness and diversity (richness and evenness) of the taxa in each group. Alpha diversity analysis, including Shannon diversity (a) and Simpson diversity (b). Principal coordinate analysis (PCoA) of sample clustering results (c) and nonmetric multidimensional scale analysis (d). A: NC group, B: PC group, C: RSH group, D: RSM group, and E: RSL group.

To analyze the structure of the intestinal flora in mice by abundance and diversity, as shown in Figure 12, alpha diversity is calculated as the species richness and diversity of the gut microbial system, which includes various analytical methods. Shannon's index and Simpson's index are the main parameters reflecting alpha diversity, which can calculate the species richness and homogeneity of the microbial community at the same time when the Shannon index is larger and the Simpson index is closer to 0, which means that the richness of the sample satisfies the requirements. The results indicated that the Shannon and Simpson values differed significantly among the 5 groups. Thus, the abundance and diversity of gut microorganisms differed significantly between each group of mice.

Beta diversity analysis was performed on all samples at the genus level, and the OTU abundance matrix of the 5 groups of samples was analyzed using principal coordinate analysis (PCoA) to downscale and simplify the data. The contribution of PC 1 was found to be 22.21% and that of PC 2 was 11.16%. PCoA scores showed that the PC group was significantly separated from the different doses of KRS treatment groups. NMDS is a clearer way to describe the distribution of flora. The results showed that the NC and KRS treatment groups were far apart, and it is presumed that KRS affects the gut microbiota of T2DM mice.

**Figure 13:**
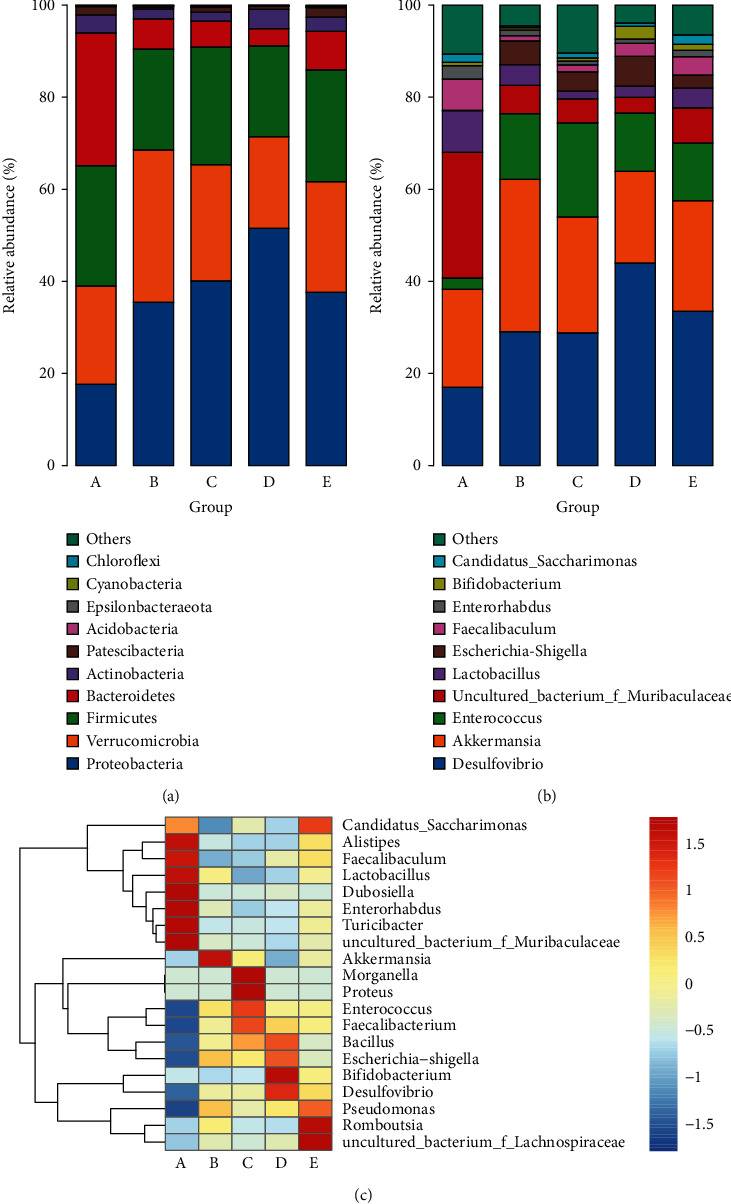
Analysis of phyla abundance in each group: (a) relative abundance distribution of operational taxonomic unit (OTU) sequences at the phylum level from the 10 most prominent bacterial phylotypes; (b) relative abundance distribution of OTU sequences at the genus level from the 10 most prominent bacterial phylotypes; (c) comparisons of gut metagenomic profiles at the genus level on a heat map. A: NC group, B: PC group, C: RSH group, D: RSM group, and E: RSL group.

According to the results of species annotation, the relative abundance of species differed significantly at the level of phylum, class, order, family, and genus from different groups, and the relative abundance at the level of phylum and genus is shown in Figure 13. At the phylum level, *Proteobacteria*, *Verrucomicrobia*, *Firmicutes*, and *Bacteroidetes* constituted the main phylum of each group, but with different compositions. Compared with the NC group (17.65%), the abundance of *Proteobacteria* in the RSH, RSM, and RSL treatment groups (40.11%, 51.48%, and 37.61%, respectively) increased significantly. In contrast, *Verrucomicrobia* abundance in the NC group (21.35%) was lower than that in the other groups except for the RSM group (19.95%). The abundances of *Firmicutes* in the PC group (21.89%) were lower than those in the NC, RSH, and RSL groups (26.12%, 25.55%, and 24.31%, respectively), while the abundances of *Bacteroidetes* (6.56%) were increased compared with NC and RSL groups (28.85% and 8.35%, respectively).

At the genus level, while the percentages of *Akkermansia*, *Desulfovibrio*, *Enterococcus*, *uncultured_bacterium_of_Muribaculaceae*, *Lactobacillus*, *Escherichia-Shigella*, *Faecalibaculum*, and *Bifidobacterium* changed significantly after the treatment of KRS, the abundance of *Enterorhabdus* and *Candidatus_Saccharimonas* did not change much. The abundances of *Akkermansia* from *Verrucomicrobia* phylum in the PC group (33.08%) were higher than those in the other groups, while the abundances of *Desulfovibrio* from *Proteobacteria* (29.09%) were decreased compared with RSM and RSL groups (43.94% and 33.51%, respectively). In the cluster analysis of the 20 bacterial genera with the highest abundance, the distribution of these genera in the KRS treatment groups at different doses was found to be significantly different from that in the PC group. In addition, there were significant differences in gut microbiota between the KRS treatment groups.

## 4. Discussion

T2DM is a metabolic disease characterized by hyperglycemia, and the prevalence of T2DM is becoming a global public health concern in the 21st century [24]. Weight loss is the main symptom of T2DM, and experimental results show that KRS treatment restores body weight in T2DM mice. Resistant starch is not digested in the gastrointestinal tract and is deposited within the digestive tract, reducing the rate of energy expenditure [25]. Some researchers have also found that the intake of resistant starch stimulates the secretion of progastrointestinal hormones and the amount of food eaten decreases, thus achieving weight control [26]. FBG and OGTT are the most direct indicators for evaluating blood glucose in T2DM patients, and changes in glucose levels trigger a feedback mechanism that promotes insulin secretion so that glucose levels in the body are always maintained within the normal range [27]. The experimental results showed that KRS can effectively reduce blood glucose level and has good restoring ability of blood glucose regulation, among which the RSM group was the most significant. Thus, these results indicated that the appropriate dose of KRS has a significant inhibitory effect on hyperglycemia. There is a strong link between lipid metabolism and glucose metabolism, and the inability to keep lipid levels within target ranges over time is a major cause of chronic complications in T2DM [28]. Therefore, the regulation of lipid metabolism is an important part of the glucose-regulating function of KRS. Typical features of dyslipidemia include increased serum concentrations of TC, TG, and LDL-C and decreased HDL-C concentrations. The experimental results showed that KRS could effectively alleviate dyslipidemia in T2DM mice, in which TC, TG, and LDL-C were significantly reduced in the RSH group, and HDL-C was significantly increased in the RSM group. HDL-C can regulate body fat metabolism and promote cholesterol excretion into the bile in addition to its antioxidant function [29]. Therefore, KRS may reduce excessive lipid deposition in liver tissues by accelerating lipid metabolism in T2DM mice and maintaining normal lipid metabolism, which plays an effective protective role for the liver.

The manifestations of T2DM include not only abnormalities of blood glucose and lipids; it is also well known that the danger is mainly reflected in the complications of T2DM [30]. Insulin resistance is a critical symptom of T2DM and a pivotal pathological basis for T2DM, obesity, and other metabolic diseases [31]. The present study was aimed at verifying the alleviating effect of resistant starch on various indexes in T2DM mice. The results demonstrated the presence of severe insulin resistance in T2DM mice by measuring serum insulin levels and determining the HOMR-IR index. The IRS-1/PI3K/Akt signaling pathway is the main pathway of insulin signaling, which regulates the expression of downstream substrate receptors and glycogen synthesis-related enzymes to maintain glucose homeostasis [32]. The possible molecular mechanisms by which KRS improves the gene level of T2DM were investigated by western blotting analysis. By binding to cell surface receptors, insulin stimulates the intrinsic kinase activity of insulin receptors, leading to autophosphorylation of insulin receptors, and the phosphorylated receptors are linked to IRS-1, which continuously transmits insulin signals and promotes phosphorylation of Akt by stimulating PI3K expression, ultimately accelerating glycogen synthesis [33]. In the current study, the addition of KRS to the diet significantly increased the protein levels of IRS-1, p-PI3K, and p-Akt in the liver. However, the total levels of PI3K and Akt were rarely affected. This result is consistent with its effect on insulin resistance. Another study using a genome-wide transcriptional approach found that resistant starch increased the expression of IRS-1 and related genes, these key genes that promote insulin secretion and insulin signaling in T2DM mice [20]). It might therefore be a key factor in the role of KRS in T2DM. Subsequently, resistant starch regulates gene expression of downstream key receptors and essential glycolytic- (uptake, synthesis, xenobiotic, and transport) related enzymes through the IRS-1/PI3K/Akt insulin signaling pathway [34]. This insulin signaling pathway is dependent on a series of intermediate pathways, including IRS, PI3K, and AKT, which are essential regulators of Glut4 expression and localization, thereby regulating cellular glucose uptake [35]. KRS treatment increased the expression of Glut4 protein, increasing glucose uptake by increasing transporter activity. Taken together, the remission of T2DM may be related to the treatment of KRS on the hepatic IRS-1/PI3K/AKT/Glut4 pathway.

Inflammatory response is important in the pathogenesis of T2DM;, TNF-*α* and MCP-1 reduce insulin-mediated glucose transport through multiple pathways; MCP-1 is a key chemokine for macrophage infiltration into adipose and liver tissues and is a potential pathogenic mechanism for the pathogenesis of low-grade chronic inflammation in this tissue; inhibition of MCP-1 expression inhibits the development of inflammation and restores insulin receptor substrate activation [36, 37]. When insulin resistance occurs in the organism, the levels of these inflammatory factors increase significantly [38]. The results of this experiment showed that in the T2DM mice, insulin resistance could delay the progression of T2DM by promoting the expression of TNF-*α* and MCP-1 inflammatory factors, while KRS treatment significantly inhibited the upregulation of inflammatory factors. Therefore, it can be inferred that KRS can control the insulin resistance status of T2DM mice, thus delaying the process of T2DM, and this effect has a certain quantity-effect relationship.

The gut microbiota is involved in various physiological activities of the body, including nutrient absorption, metabolism, and immune regulation [39]. The imbalance of gut microbiota can damage the intestinal barrier, which in turn induces systemic inflammatory responses and disorders of glucolipid metabolism [40, 41]. Once the gut barrier is disrupted, the intestinal permeability could increase and result in translocation of gut microbiota and metabolites of gut microbiota [42]. It was found that the amount of endotoxin and D-mannitol entering the blood was significantly reduced after KRS treatment of T2DM mice, indicating that KRS can effectively alleviate the damage of intestinal barrier function in T2DM. The intestinal barrier function is a critical part of the defense system. Intestinal tight junction proteins are the backbone connecting adjacent cells, controlling the integrity of the intestinal epithelial barrier, and are the most important structures regulating intestinal mucosal permeability [43]. The main role of mucins is to effectively resist the invasion of small harmful substances and pathogenic microorganisms and participate in antigen presentation, antibody and anti-inflammatory factor activation, lymphocyte activation, and cell killing, which are crucial parts of intestinal mucosal defense [44]. The intestinal defense system of T2DM mice is impaired, the expression of intestinal tight junction protein is decreased, and the gene expression of mucins is reduced. The expression of ZO-1, Occludin protein, and MUC 2 protein was increased after KRS treatment with a quantitative effect relationship; thus, KRS could normalize the expression associated with the level of intestinal mucosal protective proteins.

KRS has a better function of regulating the T2DM-induced reduction of the microbiota species and abundance in the cecum contents of mice. KRS-treated mice can enhance the content of beneficial bacteria and decrease the content of pathogenic bacteria compared with T2DM model mice. At the phylum level, the number of *Bacteroidetes* and *Firmicutes* in the intestine of T2DM mice decreased significantly, especially the percentage of *Bacteroidetes* which decreased to 6.56%, and KRS treatment increased the abundance of *Firmicutes* and *Bacteroidetes*, which are the genera directly acting on SCFAs and can effectively increase the production of SCFAs. Among them, compared to the PC group, the abundance of *Firmicutes* was significantly increased especially in the RSL group and this is consistent with previous reports [45]. The results of SCFA content showed that both propionic acid and butyric acid contents were significantly increased after KRS treatment, which was directly related to the increase of abundance of *Firmicutes* and *Bacteroidetes*. However, the acetic acid content decreased in all KRS treatment groups, which may be related to the abundance of *Lachnospiraceae* in *Firmicutes* [46]. SCFAs could promote cholesterol metabolism, and the content of butyric acid was negatively correlated with the content of lipids [47]. Moreover, *Proteobacteria* is a key factor in LPS production, and the increased abundance of LPS-producing pathogens in T2DM mice was associated with the high-fat diet during the experiment [48]. In addition, KRS treatment inhibited the abundance of *Verrucomicrobia*, leading to restoring the health structure of gut microbiota. It indicated that KRS treatment may be a potent therapeutic approach for diabetic mitigation, which contributes to the regulation of blood glucose function in T2DM mice. Furthermore, it is necessary to point out that the *Bifidobacterium* was significantly enriched in KRS supplementation in T2DM mice. *Bifidobacterium* acts as a probiotic to reduce gastrointestinal infection and alleviate inflammation, thereby promoting gut health [49]. *Escherichia-Shigella* was associated with inflammation, gut microbiota disorders, and glucose metabolism, which showed a certain positive correlation [50]. *Escherichia-Shigella* was increased in the PC group, but KRS treatment effectively reversed this change especially in low dose. These results revealed that KRS treatment could ameliorate T2DM by reversing diabetes-related changes of gut microbiota in mice.

In conclusion, KRS can reduce hyperglycemia, hyperlipidemia, and chronic systemic inflammation; improve insulin resistance and abnormal glucose tolerance; and promote hepatic glycogen synthesis in T2DM mice. The mechanism is related to the regulation of the IRS-1/PI3K/AKT/Glut4 pathway. The results of 16s rRNA gene sequencing analysis showed that treatment with KRS resulted in significant changes in the abundance and structure of the intestinal microbiota in T2DM mice and that KRS demonstrated different activities in diversifying the intestinal microbiota. These results suggest that KRS may be a potential functional food for the prevention and treatment of T2DM.

## Figures and Tables

**Table 1:** The primers for reaction.

Gene	Forward primer and reverse primer
MUC 2	F: 5′ ATGACCCAGGATGGTATCTTC 3′
	R: 5′ GTGACTGTAGTGGTGGTAATG 3′
GAPDH	F: 5′ ATCACTGCCACCCAGAAG 3′
	R: 5′ TCCACGACGGACACATTG 3′

## Data Availability

The data used to support the findings of this study are available from the corresponding author upon request.
